# Promoting Effect of Foliage Sprayed Zinc Sulfate on Accumulation of Sugar and Phenolics in Berries of *Vitis vinifera* cv. Merlot Growing on Zinc Deficient Soil

**DOI:** 10.3390/molecules20022536

**Published:** 2015-02-02

**Authors:** Chang-Zheng Song, Mei-Ying Liu, Jiang-Fei Meng, Ming Chi, Zhu-Mei Xi, Zhen-Wen Zhang

**Affiliations:** 1 College of Enology, Northwest A&F University, Yangling 712100, Shaanxi, China; E-Mails: scz1103@gmail.com (C.-Z.S.); qq493235763@163.com (M.-Y.L.); mjfwine@nwafu.edu.cn (J.-F.M.); chiming101@163.com (M.C.); 2 Shaanxi Engineering Research Center for Viti-Viniculture, Yangling 712100, Shaanxi, China

**Keywords:** zinc sulfate, foliage spray, sugar, phenolics, gene expression, grape berry

## Abstract

The effect of foliage sprayed zinc sulfate on berry development of *Vitis vinifera* cv. Merlot growing on arid zone Zn-deficient soils was investigated over two consecutive seasons, 2013 and 2014. Initial zinc concentration in soil and vines, photosynthesis at three berry developmental stages, berry weight, content of total soluble solids, titratable acidity, phenolics and expression of phenolics biosynthetic pathway genes throughout the stages were measured. Foliage sprayed zinc sulfate showed promoting effects on photosynthesis and berry development of vines and the promotion mainly occurred from veraison to maturation. Zn treatments enhanced the accumulation of total soluble solids, total phenols, flavonoids, flavanols, tannins and anthocyanins in berry skin, decreasing the concentration of titratable acidity. Furthermore, foliage sprayed zinc sulfate could significantly influence the expression of phenolics biosynthetic pathway genes throughout berry development, and the results of expression analysis supported the promotion of Zn treatments on phenolics accumulation. This research is the first comprehensive and detailed study about the effect of foliage sprayed Zn fertilizer on grape berry development, phenolics accumulation and gene expression in berry skin, providing a basis for improving the quality of grape and wine in Zn-deficient areas.

## 1. Introduction

Zinc (Zn) is an essential micronutrient for the normal healthy growth and reproduction of plants, animals and humans [1]. In plants, Zn plays a key role as a structural constituent or regulatory co-factor of a wide range of different enzymes and proteins in many important biochemical pathways. These roles include carbohydrate metabolism (both in photosynthesis and in the conversion of sugars to starch), protein metabolism, auxin (growth regulator) metabolism, pollen formation, the maintenance of the integrity of biological membranes, the resistance to infection by certain pathogens [2,3]. Therefore, many important physiological functions of Zn are unable to operate normally in Zn-deficient plants, and plant growth would be adversely affected [4].

In fact, Zn deficiency appears to be the most widespread, frequent micronutrient deficiency problem in crop and pasture plants worldwide, resulting in severe losses in yield and nutritional quality. According to a soil survey in China, it is estimated that 48.6 Mha (51.1% of farmland) is potentially Zn-deficient; available-Zn contents in calcareous soils of northern China are much lower than those in acid soils of southern China [3]. Correspondingly, related studies indicate that the effectiveness of Zn in calcareous soils of northern China is low, and Zn deficiency or serious Zn deficiency in these soils is quite common [5]. Grape is highly sensitive to Zn deficiency [3]. Visible symptoms of Zn-deficiency in grapevines are chlorosis, necrotic spots, contraction of the plant, and little leaf [3,6]. Meanwhile, the main grapevine growing areas in China are distributed in the north China [7]. Therefore, available-Zn contents in soils of the main grapevine growing areas of China are relatively low. The eastern foot of Helan Mountain of Ningxia, where the soil is mainly composed of light sierozem, is recognized as one of the best wine regions in China. Despite excellent air and water permeability, strong basicity and high pH of the soil result in the low availability of nutrient elements, especially microelements. Previous research also showed the low available content of nutrient elements, including Zn, in this area [8,9].

Microelements can significantly affect fruit quality; they can noticeably improve the quality of fruit. As one of the microelements which are strongly linked to the constitution of various proteins and physiological metabolism process, Zn also significantly affects the growth and quality of agricultural products. Many related studies have demonstrated that the application of Zn fertilizer can enhance the quality of various fruits and vegetables [10,11,12,13], and grape is undoubtedly included in this group. A study by Yamdagni *et al.* [14] showed that foliage spraying zinc sulfate could remarkably increase the soluble solid content of Thompson Seedless. Other researches also obtained similar results [15,16,17,18].

Up to now, the effect of Zn fertilizer on grape quality has provoked a number of investigations, but most of the studies focused on the content of soluble solids, acid, protein and vitamin C in grapes. Nevertheless, there are few reports concerning the influence of Zn on other flavor substances in grape, such as phenolics. Results of a recent study [12] indicated that foliar application of zinc sulfate was capable to enhance the phenolic compounds in olive fruits in semiarid areas. Furthermore, it has not yet been elucidated how phenolics biosynthesis pathway genes and related regulatory genes respond to different concentrations of foliage sprayed zinc sulfate. 

In this study, three concentration of zinc sulfate were sprayed on Merlot vines in a commercial vineyard on the Yuquanying farm in Yinchuan (Ningxia, China). Previously, soil constituents and initial concentration of Zn in grape petiole were determined to confirm the Zn-deficiency symptoms of control vines. To clarify the mechanism of phenolics biosynthesis in response to foliage sprayed zinc sulfate, photosynthetic indexes of vines at three berry developmental stages, berry weight, total soluble solids, pH and titratable acidity of developing berries, phenolics accumulation as well as fold change of gene expression in the phenolics biosynthetic pathway in berry skin throughout the developmental stages were measured. 

## 2. Results and Discussion

### 2.1. The Soil Constituents of Experimental Field Site and Initial Concentration of Zinc in Grape Petiole

Under conventional cultivation management, soil constituents change quite slowly, so soil samples were only collected in 2013, and the resulting soil constituents data are shown in Table 1. Concentrations of available N, P, K and Cu were all within the normal range, while those of Zn, Fe and Mn were lower than the critical values. The high pH of the soil coincides with the results of previous research [8,9]. The concentration of Zn in 0–20 cm and 20–40 cm soil were at low and very low levels, respectively, indicating the deficiency of available Zn in the soil of the experimental field site. In grape petioles, the critical value range of Zn in petioles is 35–50 mg·Zn·kg^−1^ dry weight [3]. The average concentration of petioles prior to treatment was 40.11 mg·Zn·kg^−1^ dry weight (Table S1), and it showed the existing of potential Zn-deficiency in the vines.

**Table 1:** Soil constituents of the experimental field site.

Soil Constituents	Depth		Critical Value
	0–20 cm	20–40 cm	
Available N (mg/kg)	34.29	17.50	10–100
Available P (mg/kg)	54.42	20.05	18–25
Available K (mg/kg)	75.19	67.78	60–100
Organic matter (%)	0.59	0.43	1–3.5
pH	8.63	8.74	-
Available Cu (mg/kg)	0.81	0.49	0.5–1.0
Available Zn (mg/kg)	0.81	0.33	1.1–2.0
Available Fe (mg/kg)	4.19	3.33	4.5–10.0
Available Mn (mg/kg)	6.52	5.17	9–15

Critical values refer to the standard values in the book by Bao [19].

### 2.2. The Photosynthesis at Three Developmental Stages

**Figure 1:**
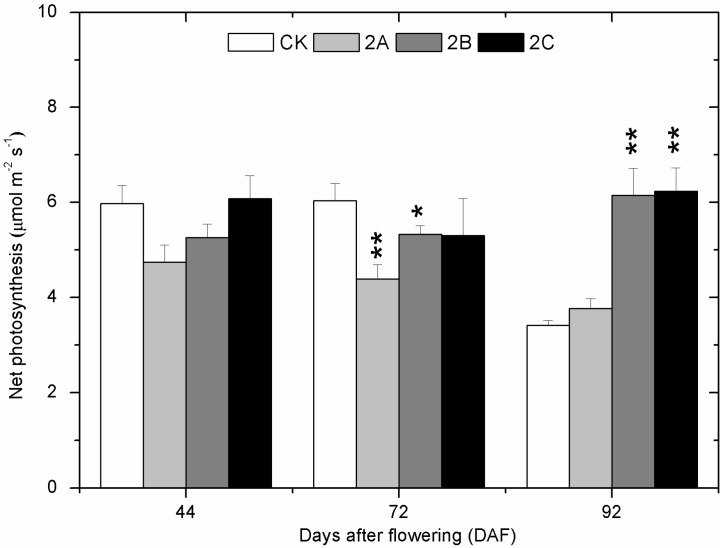
Net photosynthetic rate (Pn) of leaves at three berry developmental stages in 2013. Veraison stage corresponds to 61 DAF. The asterisk represents the Pn in the zinc treatment group is significantly different than the control group at 1% level (for two asterisks) and 5% level (for one asterisk), respectively. Statistically significant difference between the Zn-treated groups and the control was calculated by Least-significant difference (LSD) analysis. The bars indicate the mean of three values and their standard deviation.

On 44 days after flowering (DAF), the photosynthesis of CK (control) vines was higher than that of 2A (1.0 g/L of ZnSO_4_•7H_2_O treatment twice) and 2B (4.0 g/L of ZnSO_4_•7H_2_O treatment twice) vines and lower than 2C (8.0 g/L of ZnSO_4_•7H_2_O treatment twice) vines, but no significant difference was found (Figure 1). The photosynthesis of all Zn-treated vines was all lower than control vines on 72 DAF, although the difference was not significant for 2C. On the contrary, photosynthesis of all Zn-treated vines exceeded the control vines on 92 DAF, and the difference for 2B and 2C was significant. At all the three stages, the photosynthesis of Zn-treated vines increased with the concentration of the treatment.

### 2.3. Berry Weight, Total Soluble Solids, pH and Titratable Acidity of Berry Juice throughout the Developmental Stages

**Figure 2:**
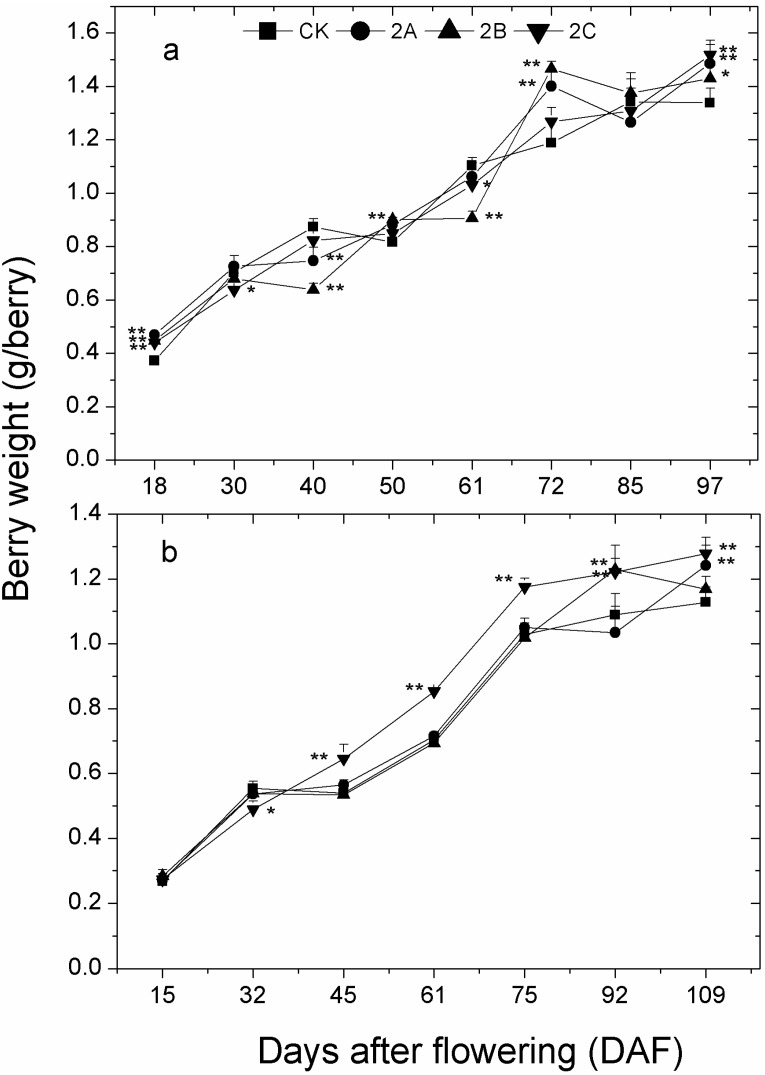
The change of berry weight with developmental stages in 2013 (**a**) and 2014 (**b**). Veraison stage corresponds to 61 DAF in both years, the same in the following figures. The symbols and bars indicate the mean of three values and their standard deviation. The asterisk represents the weight of berries in the zinc treatment group is significantly different than the control group at 1% level (for two asterisks) and 5% level (for one asterisk), respectively.

The change of berry weight experienced two fast increases at the setting and veraison stages in both 2013 and 2014 (Figure 2). In spite of several significant differences, no stable difference was found between berry weight of Zn-treated groups and that of control group before veraison (61 days after flowering (61DAF)). Notably, tendencies were observed that the increase of weight for Zn-treated berries was larger than that of control berries after veraison, and weights of Zn-treated berries, except for 2B in 2014, were significantly higher than those of control berries at maturation stage in both years. In addition, berry weight in 2C was always significantly higher than CK from 45 DAF onwards in 2014.

The contents of total soluble solids (TSS) of developing berries in 2014 and 2013 are shown in Table 2. The TSS in Zn-treated groups exceeded that of control from postveraison to maturation, and most of the differences were significant during this period. Due to the relatively later veraison in 2014 than in 2013, the promotion effects of treatment were not found until 92 DAF. The Table 3 shows the change of total pH and titratable acidity (TA) of developing berry in 2014. The differences of pH also mainly occurred after veraison, but the differences were not consistent at different sampling stages. The TA of all samples of treatments was lower than that of control from postveraison to maturation. Correlation analysis showed a significant correlation between TSS and TA (correlation = −0.975, *p* < 0.01), coinciding with the higher TSS and lower TA of Zn-treated berries between veraison and maturation.

**Table 2:** The total soluble solids (°Brix) of developing berries in 2013 and 2014.

DAF	2013				DAF	2014			
	CK	2A	2B	2C		CK	2A	2B	2C
18	3.9 ± 0.07	3.4 ± 0.04 **	3.6 ± 0.06 *	3.8 ± 0.07	15	3.4 ± 0.12	2.9 ± 0.23 *	3.1 ± 0.11	3.2 ± 0.09
30	4.0 ± 0.05	3.6 ± 0.14 *	3.6 ± 0.07 *	3.7 ± 0.07 *	32	4.1 ± 0.11	3.8 ± 0.20	4.0 ± 0.09	4.0 ± 0.13
40	3.9 ± 0.08	3.8 ± 0.10	3.7 ± 0.07	3.8 ± 0.12	45	4.2 ± 0.21	4.0 ± 0.08	4.0 ± 0.21	4.1 ± 0.03
50	5.5 ± 0.06	5.4 ± 0.06	5.7 ± 0.07 *	6.1 ± 0.06 **	61	12.5 ± 0.14	11.3 ± 0.17 **	11.7 ± 0.07 **	11.1 ± 0.18 **
61	12.4 ± 0.13	12.7 ± 0.08	12.6 ± 0.06	13.0 ± 0.00 **	75	14.7 ± 0.08	14.4 ± 0.05 **	14.6 ± 0.09	15.0 ± 0.18
72	15.4 ± 0.15	16.5 ± 0.07 **	16.3 ± 0.22 **	16.1 ± 0.20 *	92	18.9 ± 0.16	19.1 ± 0.20	19.9 ± 0.05 **	20.3 ± 0.22 **
85	17.9 ± 0.08	18.5 ± 0.19	19.0 ± 0.18 **	18.9 ± 0.09 **	109	20.1 ± 0.09	22.0 ± 0.16 **	21.8 ± 0.10 **	22.2 ± 0.12 **
97	19.6 ± 0.14	21.4 ± 0.34 **	21.6 ± 0.11 **	20.7 ± 0.21 *	-	-	-	-	-

Veraison stage corresponds to 61 DAF in both years. The asterisk represents the total soluble solids of berries in the zinc treatment group is significantly different than the control group at 1% level (for two asterisks) and 5% level (for one asterisk), respectively.

**Table 3:** The pH and titratable acidity of berries at the development stages in 2014.

DAF	pH				TA (g/L)			
	CK	2A	2B	2C	CK	2A	2B	2C
32	2.63 ± 0.021	2.66 ± 0.014	2.68 ± 0.014 **	2.66 ± 0.006	28.08 ± 0.29	27.38 ± 0.17	27.66 ± 0.12	26.91 ± 0.23 *
45	2.57 ± 0.057	2.55 ± 0.021	2.52 ± 0.005	2.56 ± 0.007	27.91 ± 0.01	27.78 ± 0.04 *	28.41 ± 0.23	27.80 ± 0.04
61	2.89 ± 0.014	3.06 ± 0.028 **	3.08 ± 0.028 **	3.05 ± 0.014 **	11.75 ± 0.29	12.61 ± 0.10	12.23 ± 0.08	12.87 ± 0.04 **
75	3.24 ± 0.007	3.31 ± 0.021 **	3.27 ± 0.014	3.26 ± 0.012	7.38 ± 0.09	7.23 ± 0.12 **	6.71 ± 0.09	7.23 ± 0.09
92	3.62 ± 0.006	3.70 ± 0.014 **	3.55 ± 0.005 **	3.66 ± 0.007 *	5.08 ± 0.11	4.44 ± 0.06	4.35 ± 0.19	4.33 ± 0.01 **
109	3.90 ± 0.007	3.86 ± 0.008 **	3.80 ± 0.007 **	3.77 ± 0.005 **	4.09 ± 0.02	3.75 ± 0.02	3.85 ± 0.04	3.63 ± 0.11 **

Veraison stage corresponds to 61 DAF in both years. The asterisk represents the pH or TA of berries in the zinc treatment group is significantly different than the control group at 1% level (for two asterisks) and 5% level (for one asterisk), respectively.

### 2.4. The Accumulation of Phenolics during Berry Development

**Figure 3:**
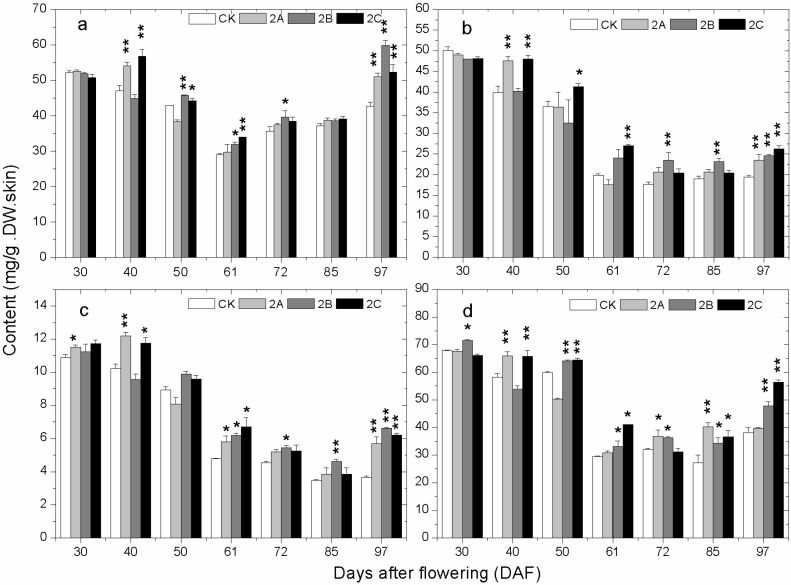
The change of (**a**) total polyphenol; (**b**) total flavonoids; (**c**) total flavanols; and (**d**) condensed tannins content in grape skin with developmental stages in the control and the Zn-treated groups in 2013. The asterisk represents the content in the zinc treatment group is significantly different than the control group at 1% level (for two asterisks) and 5% level (for one asterisk), respectively.

The changes of total phenolics (TP), total flavonoids (TFO), total flavanols (TFA) and tannins (TAN) content in grape skin at different developmental stages in 2013 are shown in Figure 3. There was no stable difference in TP among the different groups from 30 DAF to 50 DAF. Afterward, the TP of all the Zn-treated groups became higher than that of the control group. The accumulation pattern of TFO, TFA and TAN were quite similar. All of them experienced a process of decline before veraison and then became stable until a small increase on 97 DAF. At early stages, there were differences but no consistent link between the experimental and control groups. From 61 DAF to 97 DAF, the content of TFO, TFA and TAN in Zn-treated groups were generally higher than that of the control group.

**Figure 4:**
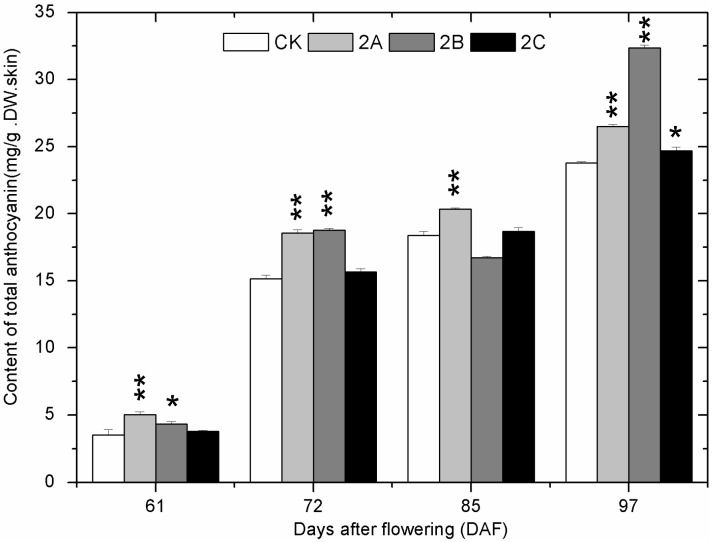
The change of total anthocyanins content in grape skin with developmental stages in the control and Zn-treated groups in 2013. The asterisk represents the content in the zinc treatment group is significantly different than the control group at 1% level (for two asterisks) and 5% level (for one asterisk), respectively.

The total anthocyanins (TAC) in grape skin could not be detected prior to veraison in all groups. From 61 DAF to 72 DAF, synthesis of anthocyanins started and the content increased rapidly (Figure 4). Then, the content continued to increase slowly until the maturation stage. The TAC of most Zn-treated groups was higher than that of the control group at all stages.

**Figure 5:**
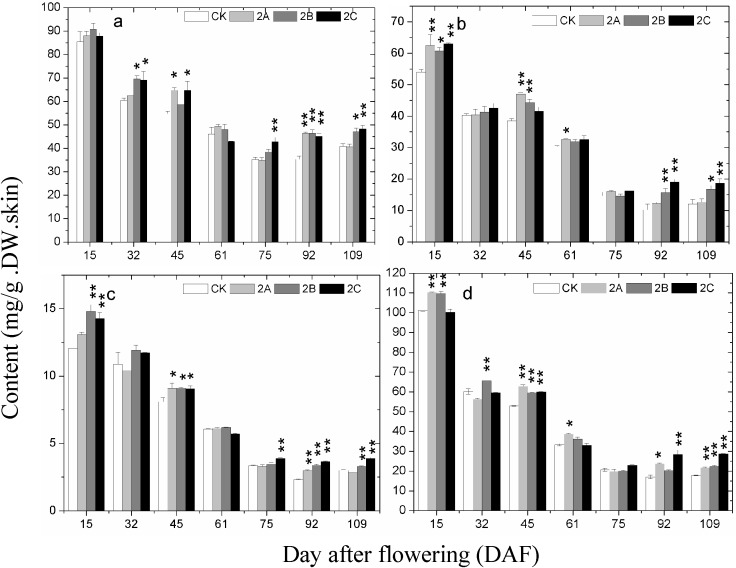
The change of (**a**) total polyphenol; (**b**) total flavonoids; (**c**) total flavanols; and (**d**) condensed tannins content in grape skin with developmental stages in the control and the Zn-treated groups in 2014. The asterisk represents the content in the zinc treatment group is significantly different than the control group at 1% level (for two asterisks) and 5% level (for one asterisk), respectively.

**Figure 6:**
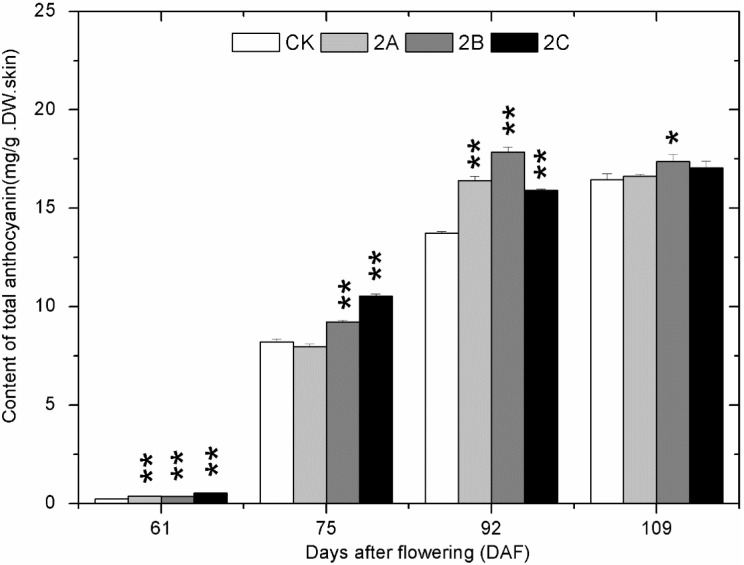
The change of total anthocyanins content in grape skin with developmental stages in the control and Zn-treated groups in 2014. The asterisk represents the content in the zinc treatment group is significantly different than the control group at 1% level (for two asterisks) and 5% level (for one asterisk), respectively.

Similar results were obtained in the repeat experiment in 2014 (Figure 5 and Figure 6). Concentrations of TP, TFO, TFA and TAN decreased slowly from 15DAF to 61DAF and then became stable from 75 DAF to 109 DAF. At the same stages (from veraison to maturation) as 2013, the phenolics contents of berry skin in most Zn-treated groups were higher than those of control. Due to the later occurrence of veraison in 2104, the concentrations of TAC in berry skin of all groups was quite low on 61DAF. Even so, the promoting effect of foliage sprayed zinc sulfate on the accumulation of TAC from veraison to maturation was quite similar to that in 2013. 2B was still the most effective treatment for accumulation of TAC in 2014. At the mature stage of both years, the content of most phenolics in berry skins of Zn-treated groups were higher than that of control group.

### 2.5. The Expression of Phenolics Biosynthetic Pathway Genes during Berry Development

**Figure 7:**
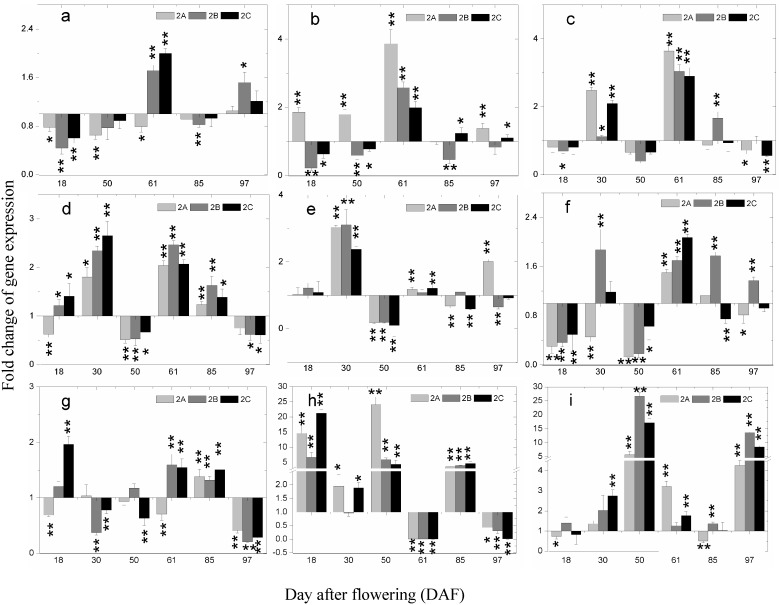
Fold changes of gene expression between the Zn-treated groups and the control at 6 developmental stages of Merlot grape berry of 2013. (**a**) *VvPAL*; (**b**) *VvSTS29*; (**c**) *VvCHS*; (**d**) *VvCHI*; (**e**) *VvF3H*; (**f**) *VvFLS4*; (**g**) *VvDFR*; (**h**) *VvLDOX*; (**i**) *VvMYBF1*. To differentiate between up-regulated expression and down-regulated expression, X axis is fixed at the position of y = 1. The bars indicate the mean of three values and their standard deviation. The asterisk represents the gene expression in the zinc treatment group is significantly different than the control group at 1% level (for two asterisks) and 5% level (for one asterisk), respectively.

To our knowledge, the expression of structural genes in the phenolics biosynthesis pathway in response to foliage spraying of Zn has not been described in previous studies. In this research, a two-fold change cutoff was used to define differentially expressed genes between the Zn-treated groups and the control group in the year of 2013. The expression of related genes (*VvPAL*, *VvSTS29*, *VvCHS*, *VvCHI*, *VvF3H*, *VvFLS4*, *VvDFR*, *VvLDOX* and *VvMYBF1*) in grape berry skin were all significantly influenced by the foliage spraying of Zn at stages of berry development (Figure 7). However, the effects on different genes at the same stage were not identical. At 18 DAF, the expression of *VvFLS4* was significantly inhibited, while *VvLDOX* was highly induced, the other genes were not notably affected. On 30 DAF, the fold change of expression of *VvPAL* and *VvSTS29* were not detected due to the tiny expression quantity. The expression of other genes, except for *VvDFR* and *VvFLS4* in the 2A treatment group, were all up-regulated. On 50 DAF, *VvLDOX* and *VvMYBF1* were highly induced. The other genes except for *VvSTS29* in 2A treatment group and *VvDFR* in 2A treatment group were all inhibited, but most of the effects were not significant. On 61 DAF, a tendency for increase in the expression of all genes was observed, except for *VvLDOX*. Conversely, only *VvLDOX* was induced drastically on 85 DAF. At the maturation stage (97 DAF), the expression of *VvDFR* and *VvLDOX* was significantly inhibited and that of *VvMYBF1* was highly induced.

### 2.6. Discussion

As an essential micronutrient, Zn plays a vital role in various plant growth and development processes, including primary and secondary metabolism [1,2,3]. Previous studies were mostly concerned the effect of Zn fertilizer on the quality of mature grape berries, and the studied quality parameters seldom included secondary metabolite like phenolics. Moreover, there were no comprehensive and detailed studies about the effect of foliage sprayed Zn fertilizer (zinc sulfate) on the whole development process of grape berries. The main results of the current research were as follows: firstly, the promotion effects of foliage sprayed zinc sulfate on grape berry development mainly occurred in the period from veraison to maturation. Secondly, Zn treatment enhanced the photosynthesis of vines at maturation, the accumulation of TSS, TP, TFO, TFA, TAN and TAC in berry skin, decreasing the concentration of TA on general trend. Finally, the markedly influenced expression of related genes could help to explain the promotion of Zn treatment on phenolics accumulation. 

The soil factors affecting the availability of Zn to plant include pH, clay content, calcium carbonate content, redox conditions, soil moisture status, concentrations of macro-nutrients like phosphorus and so on [3,20,21]. The high pH and phosphorus concentration of the soil sample coincided with the low concentration of available Zn, which led directly to the deficiency of Zn in vines seen in this study.

According to the results of the present study, we could find that most differences of the measured indexes between Zn-treated groups and control group occurred from veraison to maturation, when the primary metabolism (sugar metabolism) and secondary metabolism (phenolic metabolism) were both highly active. It suggested that foliage sprayed zinc sulfate could alleviate Zn-deficient and enhance the development of grape berry during this period. In other words, the treatment delayed the maturation and extended the time of substance accumulation.

In Zn-deficient plants, the activity of carbonic anhydrase (CA), ribulose 1,5-biphosphate carboxylase (RuBPC) and the content of chlorophyll decrease, leading to a decline of photosynthesis [3,22,23]. Meanwhile, the translocation of sucrose from leaves to fruit is also impaired [3]. The promotion effect of foliage sprayed zinc sulfate on photosynthesis of Zn-deficient vines at the late developmental stages was possibly the origin of the initiation of the change of other parameters considering the relationship between photosynthesis, sugar metabolism and phenolic metabolism.

Berry weight, determined by berry size and density, is one of the essential factors contributing to grape quality. Various practical techniques such as deficit irrigation [24,25], trunk girdling [26], light pruning and exogenous hormone [27] have been used to control berry weight. In the present study, the larger increase of berry weight in Zn-treated groups in 2013 corresponded to the higher photosynthesis at maturity. Hence, the more dry matter accumulation probably led to the higher weight. Furthermore, Zn is required for the synthesis of auxin (IAA) [3], which has been linked to elongation and division of cells [28]. The higher weight could also be related to higher content of IAA in Zn-treated berries.

Numerous studies have described the enhancing effects of foliage sprayed zinc sulfate on the content of TSS in mature fruits [14,17]. Results in this study suggested that the promotion started at veraison. Correlation analysis showed a significant correlation between TSS and berry weight in 2014 (correlation = 0.944, *p* < 0.01), verifying the higher berry weight in Zn-treated groups. The major organic acids in grape berry are tartaric, malic and citric acid, and the composition and content of these acids influence the flavor and quality of grape [29]. Although a significant correlation between pH and TA was found throughout the developing stages in current study, the lower pH values did not correspond to more TA contents in Zn-treated groups. It suggested that the treatments had different effects on the accumulation of different organic acids, leading to the change of both content and percentage of each acid.

To our knowledge, both phenolics and Zn have been the subject of a number of studies. However, very little work has been done to investigate the relationship between biosynthesis of phenolics and application of Zn fertilizer. Due to the fact sucrose is a positive regulator of the biosynthesis of phenolics, particularly flavonoids [30], the improvement of Zn treatment on photosynthesis and sugar accumulation could possibly enhance the biosynthesis of flavonoids. In this study, a notably promoting effect of foliage sprayed zinc sulfate on the accumulation of phenolics during berry development was found in both 2013 and 2014.

Total phenolics (TP), total flavonoids (TFO), total flavanols (TFA), tannins (TAN) and total anthocyanins (TAC) are important indicators in grape and wine, and their content determines the quality of grape and wine. At early stages, the contents of TP, TFO, TFA and TAN decreased continuously. The results coincided with previous studies [31,32]. At veraison, a rapid decline happened to the content of TP, TFO, TFA, and TAN. Meanwhile, most of the contents of these phenolic compounds in Zn-treated groups exceeded those of the control group. Besides, synthesis of anthocyanins started at this stage, and the contents in 2A, 2B and 2C groups were mostly higher than those in the control group. Combined with the analysis of gene expression at 61 DAF, the results suggested that the promotion of phenolic compound biosynthesis in Zn application groups could be caused by the higher expression of genes in phenolics biosynthetic pathway at veraison. Furthermore, flavanols are some of the metabolites of the anthocyanin biosynthetic pathway; at veraison, upstream enzymes in the flavanols biosynthetic pathway are likely to flow into the anthocyanidins biosynthetic pathway [33]. Therefore, the more transformation of flavanols into anthocyanidins may also cause the higher content of TAC in Zn-treated groups. From postveraison to maturation, content of TP, TFO, TFA, TAN and TAC in Zn-treated groups kept higher than that of control. At maturation, nearly all the differences were significant. The small differences in the accumulation of phenolics observed in the samples of 2013 and 2014 could have arisen not only from climate conditions but also from the fact that the vines had a previous Zn application in 2014 that could have marginally affected the results due to a cumulative effect.

These results of both years indicated that the range of 1.0 g/L–8.0 g/L foliar application of ZnSO_4_•7H_2_O could effectively enhance the accumulation of phenolic compounds in grape berries. Previous studies on other crops were also carried out using the same range of ZnSO_4_•7H_2_O concentrations [11,12,34].

The genes investigated in present study are key genes in the phenolics biosynthetic pathway, which are generally used in related studies [35,36,37]. Expression analysis of these genes along berry development could reveal the mechanisms of phenolics accumulation in response to foliar application of ZnSO_4_•7H_2_O. PAL is the first key enzyme in phenolics biosynthesis pathway. In strawberries, red grapes, apples and pears, PAL activity has two peaks during development, one during the early development of the fruit, and the other at maturity. Interestingly, the expression of *VvPAL* was up regulated at 61 DAF and 97 DAF, the stages just after *VvPAL* was highly expressed. In other words, zinc sulfate treatment inhibited the decrease of *VvPAL* expression level on 61 DAF and 97 DAF. This could possibly explain the smaller decrease of TP, TFO, TFA and TAN on 61 DAF and bigger increase of TP, TFO, TFA, TAN and TAC on 97 DAF. Stilbene synthase (STS) catalyzes the biosynthesis of stilbenic compounds [38]. The biochemical reaction catalyzed by STS follows that catalyzed by PAL. Therefore, the expression pattern and induced response of *VvSTS29* were quite similar to those of *VvPAL*. In the present study similar results were obtained. 

CHS and CHI are key enzymes in the flavonoid biosynthesis [39]. Previous studies demonstrated that over-expression of the *VvCHI* gene enhanced flavonoid production [40]. Besides, the *VvF3H* gene represents a pivotal point in the regulation of flavonoid biosynthesis because its expression is coordinated with many subsets of genes in various plant species [41]. The three genes identically responded to foliage spraying of Zn in this study. The induced expression of these genes may also contribute to the higher flavonoids content in grape berry of Zn-treated groups due to their functions.

The *VvFLS4* gene catalyses the last step of the flavonoid biosynthetic pathway, directly leading to different flavonol products. Meanwhile, MYBF1 was confirmed to be a transcription regulator of FLS4 in a recent study [42]. In this study, *VvMYBF1* was up-regulated at most stages in each Zn-treated group. Particularly on 50 DAF and 97 DAF, the expression of *VvMYBF1* was highly induced. The transcription of *VvMYBF1* in the skins of berries throughout grape berry development was highly correlated with the accumulation of flavonols. Two distinct periods of flavonol synthesis occur in grapes: around flowering and during ripening of the developing berries, respectively [43]. Hence, the high inductive effect on *VvMYBF1* (50 and 97 DAF) and *VvFLS4* (61 DAF) could lead to the higher flavonol content in berries of Zn-treated groups.

DFR and LDOX catalyze the formation of proanthocyanidins and anthocyanidins [44,45]. The inductive effect on *VvDFR* expression on 61 DAF (except for 2A treatment) and 85 DAF could help to explain the higher content of TAC in Zn-treated groups. The whole flavonoid pathway can be divided into two parts by LDOX: the basic flavonoid upstream pathway and the specific anthocyanin downstream branch [44]. At veraison it was significantly inhibited; in other words, the process of the flavonoid flux into the anthocyanin branch was suppressed in Zn-treated groups. The result may explain the higher content of TFO, TFA and TAN in berry skin of Zn-treated groups at this stage. The inductive expression of *VvLDOX* after veraison may relate to the faster accumulation and higher content of anthocyanins in all Zn-treated groups.

As shown in Figure 7, 2B and 2C treatments had more consistent effects on the expression of the tested genes throughout berry development. For those stages when different effects occurred, it was generally 2A treatment that led to different effect from the other two treatments. The result suggested that low concentration of foliage sprayed Zn may not lead to authentic effects on the expression of related genes. Furthermore, comparing the expression of these genes in response to foliage sprayed Zn, *VvLDOX* and *VvMYBF1* were the most affected. 

At veraison, various physiology and biochemistry changes happen in grape berries, such as the beginning of berry softening and coloring, and the remarkable change of proportions of different plant hormones and phenolics. In the present study, noticeable differences of both phenolics content and expression of related genes occurred between the Zn-treated groups and the control group at this stage. Considering the complexity of phenolics biosynthesis, future work will focus on larger scale (like genome-wide scale) and more detail phenolics composition in berry skin of Merlot grown under deficient, sufficient and excess supply of Zn.

## 3. Experimental Section

### 3.1. Experimental Field Site

Vine (*Vitis Vinifera* cv. Merlot) treatments were carried out in a commercial vineyard (38.27 °N, 106.06 °E) on the Yuquanying farm in Yinchuan (Ningxia, China) in 2013 and 2014. The experimental site was chosen on the basis of previous reports of Zn-deficiency in the Ningxia region [8,9]. Considering the influence of soil conditions on vine Zn status, the Ningxia region has a high probability of developing Zn-deficiency given its typical soil type, that is, high pH and high salinity. And for all we know, vines at the experimental site had not previously received any foliar spraying of Zn-treatment. Soil of the experimental field was sampled according to the principle of random equivalent after the harvest and before autumn fertilizing. The soil samples were air-dried and sent to Soil and Fertilizer Institute (Northwest A&F University, Yangling, China) for analysis in 2013. Analytical methods of soil attributes were as described in the book of Bao [19]. The analysis of Zn was conducted using graphite furnace atomic absorption spectrometry (GFAAS). Grape petioles in the opposite position of first cluster were also sampled two weeks before flowering (prior to when the first treatment was performed) to determine the initial condition of Zn concentration in untreated vines.

### 3.2. Experimental Design

The vines within the experiment, own-rooted and planted in north-south orientation in 1998, were selected on the basis of vine uniformity. The training system was vertical single cordon positioning system. Space within the vine rows was 0.6 m, and 3.0 m between the rows. No obvious visible Zn-deficiency symptom was found in the vines. The treatments were randomly applied to blocks of 3 panels (15 vines in each panel) in 6 consecutive rows (each two rows forming a replicate) using a completely randomized design. Buffer vines were arranged between treatments in each row. 

In both years, foliar sprays were applied on the Zn-treated vines using a hand-held pump sprayer. The Zn was applied in an aqueous solution of zinc sulfate (ZnSO_4_•7H_2_O) added 1% of Tween 80. As shown in Table 4, three concentrations (A: 1.0 g/L, B: 4.0 g/L and C: 8.0 g/L) of ZnSO_4_•7H_2_O was sprayed for twice, 2 weeks before flowering and 2 weeks after flowering (2A, 2B and 2C), respectively. About 0.3 L of zinc sulfate solution was sprayed in each vine on average. These spraying concentrations and times were selected based on the results obtained in previous studies [11,12,34]. Control vines were sprayed with water added 1% of Tween 80 alone.

**Table 4:** Experimental scheme of foliar sprayed Zn-treatments.

Treatments	ZnSO_4_•7H_2_O Concentration (g/L)	Spraying Time	
		2 Weeks BF	2 Weeks AF
CK	0.0	√	√
2A	1.0	√	√
2B	4.0	√	√
2C	8.0	√	√

### 3.3. Determination of Photosynthesis

Determination of photosynthesis was carried out at three stages. The dates were 19 July 2013 (44 DAF), 16 August 2013 (72 DAF) and 05 September 2013 (92 DAF). Four vines of each treatment were randomly chosen for photosynthesis determination by LI-6400 portable Photosynthetic apparatus (LICOR, Lincoln, NE, USA) at each determination date. Net photosynthetic rate (Pn) of leaves, on the third to fifth node, of the middle or upper bearing branches was determined. The determination was performed between 10:00 am and 11:00 am. Data of each leaf were read three times.

### 3.4. Berry Sample Collection

In 2013, berries were sampled once nearly every 10 days at eight developmental stages, from modified E-L stage (MELS) 31 (Berries pea size) (20 DAF) to MELS 38 (Harvest, 97 DAF). In 2014, berries were sampled once a fortnight at seven developmental stages, from 19-Jun-14 (15 DAF) to 21-Sep-14 (109 DAF). The dates were shown in Table S2. For each sampling date, a 350-berry sample was randomly collected from each replicate. The sample was collected to obtain representative berries of the vines by considering the variability of the positions of berries on bunches, and bunches on vines. Three replicates were selected from each treatment. From each replicate, 50 berries were randomly selected, enclosed in silver paper, and placed in a liquid nitrogen container. The rest of the samples were placed in foam boxes filled with ice bag. All samples were transported to the laboratory within two hours and stored at −80 °C prior to subsequent analysis.

### 3.5. Berry Weight, Total Soluble Solids, pH and Titratable Acidity of Berry Juice

The fresh weight of about 100 berries from each sample was determined and the mean berry weight was calculated by dividing the total. Subsequently those berries were juiced and total soluble solids (TSS) measured using a TD-45 digital refractometer (TOP, Zhejiang, China). The berry pH was measured using PB-10 pH meter (Sartorius, Gottingen, Germany). Titratable acidity (TA) was determined with sodium hydroxide titration.

### 3.6. Sample Preparation for Phenolics Analysis

Phenolics extraction was performed from skins of 200 frozen berries, randomly selected from each sample. Frozen berries were peeled without thawing, and the obtained skins were homogenized in liquid nitrogen with a chilled pulverizer. Then the skins were dried with a lyophilizer. To standardize the process, 1.0 g of dried skin samples were used for phenolics extraction. The samples were put into 50 mL centrifuge tubes, which were swathed in black tapes, and 20 mL hydrochloric acid methanol solution (60% methanol, 0.1% hydrochloric acid) was added. After 30 min of sonication at 40% power and 30 °C, samples were centrifuged at 10,000 rpm for 10 min at 4 °C. The supernatant liquor of each sample was collected into a 100 mL jar. The extraction was repeated three times. The three supernatants were combined and stored at −80 °C. All extraction steps were done in the dark, to prevent phenolics decomposition and oxidation.

### 3.7. Analysis of Phenolics in Skins of Grape Berries

TP content was determined by using the Folin–Ciocalteu method [46]. The TP concentration was expressed as milligrams gallic acid equivalence (GAE) per gram of dry berry skin (mg GAE/g DW). TFO content was determined according to the method of Peinado *et al*. [47]. Results are expressed as milligrams rutin equivalence (RE) per gram of dry berry skin (mg RE/g DW). TFA content was determined with *p*-DMACA [48]. Results are expressed as milligrams (+)-catechin equivalence (CE) per gram of dry berry skin (mg CE/g DW). TAC content was determined by the pH-differential method. Total anthocyanin content was expressed as milligrams of cyanidin-3-monoglucoside equivalence per gram of dry berry skin (mg ME/g DW) [49]. TAN was determined by the methyl cellulose precipitation (MCP) method [50]. And the results are expressed as milligrams (+)-catechin equivalence (CE) per gram of dry berry skin (mg CE/g DW). Statistical significance was evaluated using the Least Significant Difference test. 

### 3.8. Analysis of Transcriptional Level by Real-Time PCR

Total RNA was extracted with a Column Plant RNAout 2.0 (TIANDZ, Beijing, China) from three samples for each of the experimental conditions. All RNA samples were digested to remove genomic DNA and reverse-transcribed in a 20 μL reaction mixture for cDNA synthesis using a PrimeScript RT Reagent Kit with gDNA Eraser (Takara, Shiga, Japan) as described in the manufacturer’s manuals. The integrity of RNA was verified by resolving in 1% formaldehyde-agarose gels and subsequent ethidium bromide staining. RNA-purity was assessed based on absorbancce ratio of 1.8 to 2.0 at 260/280 nm using Nanodrop ND-1000 Spectrophotometer (Nanodrop Technologies, Rockland, DE, USA).

The expression levels of phenolics biosynthesis-related genes in berry skins were determined by quantitative real-time PCR (qRT-PCR) using a Bio-Rad IQ5 Real-Time PCR system (Bio-Rad Laboratories, Berkeley, CA, USA) and a SYBR Real-time PCR premixture (Bioteke Corporation, Beijing, China) as described in the manufacturers’ manuals. Real-time PCR reaction mixture (20 μL) was composed of 10 μL 2× Premix, 1.0 μL primers (forward primer and reverse primer, 10 uM), and 1.0 μL cDNA and 8 μL sterilized ddH2O. The template cDNA was denatured at 95 °C for 30 s followed by 45 cycles of amplification with 95 °C for 15 s, 60 °C for 20 s, 72 °C for 30 s and a melt cycle from 60 °C to 95 °C. The sequences of the primers used for real-time PCR were referred from previous works as shown in Table S3.

Data were normalized against *VvACTIN*, and the average expression level of each gene was calculated as the molar ratio relative to the copy number of *VvACTIN*. The fold changes in relative gene expression between the Zn-treated groups and the control group at the same time point were calculated using the 2^−ΔΔCT^ methods described by Livak and Schmittgen [51], where ΔΔCT = (CTtarget − CT*_VvACTIN_*)treat − (averageCTtarget − averageCT*_VvACTIN_*)control. The mean and the standard derivation (SD) were estimated after 2^−ΔΔCT^ calculation. 

### 3.9. Presentation of Results

Mean and standard deviation of values, significance of difference analysis and correlation analysis were analyzed by using software SPSS Statistics 20.0 (IBM, Armonk, NY, USA). Figures were made by using the drawing software Origin 9.0 (OriginLab, Hampton, MA, USA).

## 4. Conclusions

A comprehensive and detailed study about the effect of foliage sprayed Zn fertilizer (zinc sulfate) on the whole development process of grape berries was conducted, on arid zone Zn-deficient soils, over two consecutive seasons, 2013 and 2014. The results showed that foliage sprayed zinc sulfate could promote the photosynthesis and berry development of vines, and the promotion mainly occurred from veraison to maturation. Meanwhile, Zn treatment enhanced the accumulation of TSS, TP, TFO, TFA, TAN and TAC in berry skin, and decreased the concentration of TA on general trend. Moreover, expression of phenolics biosynthetic pathway genes throughout berry development was also significantly influenced by Zn treatment, which could help to explain the promotion effect on phenolics accumulation. The current research provides a theoretical basis for improving the quality of grape and wine in grapevine growing areas with deficient available Zn in the soil.

## Supplementary Materials

Supplementary materials can be accessed at: http://www.mdpi.com/1420-3049/20/02/2536/s1.
