# Supplementary material for: Promoting Effect of Foliage Sprayed Zinc Sulfate on Accumulation of Sugar and Phenolics in Berries of Vitis vinifera cv. Merlot Growing on Zinc Deficient Soil
Source: Molecules. 2015 Feb 2;20(2):2536–54. doi: 10.3390/molecules20022536 (PMC6272641; doi:10.3390/molecules20022536)
Supplement: Supplementary file 1 [file molecules-20-02536-s001.pdf]

Supplementary Materials

Table S1. The average concentration of petioles prior to treatment.

Sample Number	Concentration of Zinc (mg/kg DW)
1	40.46
2	50.31
3	46.38
4	37.35
5	58.85
6	39.04
7	46.73
8	43.53
9	55.01
10	48.79
11	52.27
12	52.04
13	17.35
14	58.79
15	43.89
16	32.47
17	55.72
18	38.96
19	54.94
20	38.75
21	31.24
22	28.02
23	71.90
24	50.95
25	23.48
26	14.22
27	33.63
28	44.49
29	64.35
30	56.42
31	15.23
32	12.33
33	77.60
34	3.05
35	45.02
36	12.47
37	34.25
38	19.05
39	29.08
40	38.69
41	50.83
42	37.59
43	40.13
44	46.49

Table S1. *Cont.*

Sample Number	Concentration of Zinc (mg/kg DW)
45	24.11
46	29.99
47	24.09
48	74.61
49	67.28
50	39.09
51	16.75
52	18.50
53	20.79
54	47.04
55	21.63
56	24.20
57	35.57
58	70.49
59	72.37
60	28.13
Average	40.11

Table S2. Sample collection dates and the corresponding days after flowering (DAF) in 2013 and 2014.

2013		2014	
**Date**	**DAF**	**Date**	**DAF**
23 June 2013	18	19 June 2014	15
5 July 2013	30	6 July 2014	32
15 July 2013	40	19 July 2014	45
25 July 2013	50	4 August 2014	61
5 August 2013	61	18 August 2014	75
16 August 2013	72	4 September 2014	92
29 August 2013	85	21 Septemner 2014	109
10 September 2013	97	-	-

18 DAF, 61 DAF, 97 DAF corresponded the pea size, veraison and mature stages in the year of 2013, respectively. And 15 DAF, 61 DAF, 109 DAF corresponded the pea size, veraison and mature stages in the year of 2014, respectively.

Table S3. Primers used for the quantification of gene expression levels by qRT-PCR.

Gene Name	Primer	Sequence (5’→3’)	Reference
VvPAL	F	GTTGTCGTGAAAAACCAGCTT	Höll *et al.* [1]
	R	GGATCACTCACGACGAAACTC	
VvCHS	F	GTCTGAAGGAAGAGAAACTGAGAG	Zheng *et al.* [2]
	R	CCAGGATAAACAACACGCAT	
VvSTS29	F	GGTTTTGGACCAGGCTTGACT	Höll *et a*l. [1]
	R	GAGATAAATACCTTACTCCTATTCAAC	
VvCHI	F	TGGAAGGGCAAGACTGTG	Zheng *et al.* [2]
	R	TGAATACTGGCGACCCGT	

Table S3. *Cont.*

Gene Name	Primer	Sequence (5'→3')	Reference
VvF3H	F	CTCCTACCCACTACGAACC	Zheng *et al.* [2]
	R	CAGACAACACCTCCAGCA	
VvFLS4	F	AAACCACCTACTTACAGAGC	Azuma *et al.* [3]
	R	ACCTAACCCCAGTGACAGAC	
VvMYBF1	F	GGAGGTTGAGGGGTTGTG	Azuma *et al.* [3]
	R	AAGTTGGGGAAGAGCAGGAG	
VvDFR	F	GAAACCTGTAGATGGCAGGA	Ali *et al.* [4]
	R	GGCCAAATCAAACTACCAGA	
VvLDOX	F	ACCTTCATCCTCCACAACAT	Ali *et al.* [4]
	R	GTAGAGCCTCCTGGGTCTT	
VvACTIN	F	GATTCTGGTGATGGTGTGAGT	Guo *et al.* [5]
	R	GACAATTTCCCGTTCAGCAGT	

Reference

1. Holl, J.; Vannozzi, A.; Czemmel, S.; D'Onofrio, C.; Walker, A.R.; Rausch, T.; Lucchin, M.; Boss, P.K.; Dry, I.B.; Bogs, J. The R2R3-MYB Transcription Factors MYB14 and MYB15 Regulate Stilbene Biosynthesis in Vitis vinifera. *Plant Cell* **2013**, *25*, 4135–4149.
2. Zheng, Y.; Li, J.H.; Xin, H.P.; Wang, N.; Guan, L.; Wu, B.H.; Li, S.H. Anthocyanin profile and gene expression in berry skin of two red *Vitis vinifera* grape cultivars that are sunlight dependent *versus* sunlight independent. *Aust. J. Grape Wine Res.* **2013**, *19*, 238–248.
3. Azuma, A.; Yakushiji, H.; Koshita, Y.; Kobayashi, S. Flavonoid biosynthesis-related genes in grape skin are differentially regulated by temperature and light conditions. *Planta* **2012**, *236*, 1067–1080.
4. Ali, M.B.; Howard, S.; Chen, S.; Wang, Y.; Yu, O.; Kovacs, L.G.; Qiu, W. Berry skin development in Norton grape: Distinct patterns of transcriptional regulation and flavonoid biosynthesis. *BMC Plant Biol.* **2011**, *11*, 7, doi:10.1186/1471-2229-11-7.
5. Guo, R.; Xu, X.; Carole, B.; Li, X.; Gao, M.; Zheng, Y.; Wang, X. Genome-wide identification, evolutionary and expression analysis of the aspartic protease gene superfamily in grape. *BMC Genomics* **2013**, *14*, 554, doi:10.1186/1471-2164-14-554.
